# Multisensory-Based Rehabilitation Approach: Translational Insights from Animal Models to Early Intervention

**DOI:** 10.3389/fnins.2017.00430

**Published:** 2017-07-26

**Authors:** Giulia Purpura, Giovanni Cioni, Francesca Tinelli

**Affiliations:** ^1^Department of Developmental Neuroscience, Fondazione Stella Maris (IRCCS) Pisa, Italy; ^2^Department of Clinical and Experimental Medicine, University of Pisa Pisa, Italy

**Keywords:** multisensory integration, early intervention, neurodevelopmental disabilities, multisensory rehabilitation, visual disorders

## Abstract

Multisensory processes permit combinations of several inputs, coming from different sensory systems, allowing for a coherent representation of biological events and facilitating adaptation to environment. For these reasons, their application in neurological and neuropsychological rehabilitation has been enhanced in the last decades. Recent studies on animals and human models have indicated that, on one hand multisensory integration matures gradually during post-natal life and development is closely linked to environment and experience and, on the other hand, that modality-specific information seems to do not benefit by redundancy across multiple sense modalities and is more readily perceived in unimodal than in multimodal stimulation. In this review, multisensory process development is analyzed, highlighting clinical effects in animal and human models of its manipulation for rehabilitation of sensory disorders. In addition, new methods of early intervention based on multisensory-based rehabilitation approach and their applications on different infant populations at risk of neurodevelopmental disabilities are discussed.

## Introduction

Human capacity to use different senses and combine different sources of information is key to understanding surrounding environment and gradually initiate adaptive behaviors. This synergy produces a percept whose reliability is much greater than a sum of information coming from different sensory channels and it is also a powerful asset in signal disambiguation, including human speech and animal communication. Facilitation of these competences has a large adaptive value and it is present in all extant species. The expression “Multisensory Integration” (MI) describes this neurobiological process, “by which information from different sensory systems is combined to enhance and accelerate detection, localization, and reaction to biologically significant events” (Stein et al., 2009).

This review aims to: (1) examine developmental trajectories of multisensory processes both in animal and human models; (2) highlight effects of a multisensory-based rehabilitation approach in adults and children with visual disorders; and (3) explore the potential effect of multisensory-based rehabilitation approach in the context of early intervention in children with some neurodevelopmental disabilities.

## Development of multisensory integration

The superior colliculus (SC) plays an important role in integrating information from multiple sensory channels but its multisensory properties seem to be only potential in newborns and appear gradually during postnatal life, depending on the relationship between neural networks and environmental events (Wallace and Stein, 1997). From studies on cat models, it is evident that SC neurons at birth are predominantly responsive to unisensory stimuli. In fact, the capacity to integrate concordant cross-modal cues requires months of maturation, suggesting that experience with cross-modal signals may be important in structuring operational multisensory principles (Stein et al., 2009). According to Wallace and Stein (1997), multisensory neurons first appear toward the end of the second postnatal week in the cat model. Although they are comparatively rare at this time, their incidence increases gradually over a 10–12 week period. Furthermore, Wallace et al. (2004, 2006) demonstrated that early visual experience is critical for development of MI abilities in the SC, as well as corticotectal inputs result necessary for full functional maturation of this process. To address the question of where, in the circuit underlying MI, early cross-modal experience exerts its effects, Wallace and Stein deactivated the anterior ectosylvian sulcus (AES) and the lateral suprasylvian sulcus (rLS) during adulthood (Wallace and Stein, 1994) and during the temporal window in which MI develops (Wallace and Stein, 1997) in cats. These procedures led to significant MI impairments in neurons of the ipsilateral SC. Consequently, cats were unable to combine their inputs from different sensory channels in order to enhance responses and these same deficits were evident in behavior. Jiang et al. (2006) added that removal of either AES or rLS during the neonatal period had comparatively fewer consequences on MI properties than if removal occurred in adulthood, because brain plasticity is maximal in early life.

In addition to the studies on the cat model, data from the primate brain also suggest that the capacity to synthesize multisensory information does not simply appear in SC neurons at a prescribed maturational stage but rather develops only after substantial experience with cross-modal stimuli (Wallace and Stein, 2001).

In human adults, the capacity of Central Nervous System (CNS) to combine different sensory information was widely investigated to estimate and measure the processes that improve perception when individuals integrate redundant stimuli (Ernst and Banks, 2002). More recently, findings from studies on animal models and human adult have been supplemented by evidence from behavioral and electrophysiological studies on infants and children (Neil et al., 2006; Brandwein et al., 2011; Hyde et al., 2011; Gori et al., 2012).

Neil et al. (2006) studied multisensory processing capabilities in healthy and full term infants (0–10 months old) and compared their performances with adults. According to their results, response latencies are generally faster to audiovisual targets, somewhat slower to visual targets, and slowest to auditory targets. However, in contrast to adults, only older infants exhibited evidence of such facilitation as expected in MI.

Also Lewkowicz (1996, 1998, 2000, 2008) showed that multisensory perception is central to adaptive behavior because it allows us to gradually perceive a world of coherent perceptual entities and, according to this author, 3-month-old infants can already recognize and discriminate dynamic audiovisual sequences and 4-month-old infants rely on multisensory redundancy for successful perception and discrimination. Their responses to audiovisual order changes are consistent with the fact that multisensory redundancy is particularly important for infant learning and discrimination in the early months of life (Lewkowicz, 2008).

Bremner and Spence (2008) explained as multisensory condition offers across development an enhanced, immediate and uniform representation of the environmental properties. In this perspective, Bahrick et al. (Bahrick and Lickliter, 2002; Bahrick et al., 2015) suggested that in early infancy synchronous multisensory stimulation facilitates the detection of amodal properties of events (characteristic redundantly specified across more than one sense modality, e.g., shape, duration, rhythm, and synchrony). With this in mind, the “intersensory redundancy,” that is the synchronized stimulation across multiple senses, could be pivotal in early perceptual, cognitive, and social development. On the other hand, perception of modality-specific information (e.g., color, orientation, auditory pitch, and timbre) seems to do not benefit by redundance across multiple sense modalities, while it is faster with unimodal than multimodal stimulation. In fact, the capacity of detecting both amodal and modality-specific properties of events is possible later in development, as attention becomes more flexible and processing becomes more efficient (Bahrick et al., 2006, 2010).

According to this line of research, maturation of multisensory processes starts very early in life, but fully develops in adolescence. Recently, results of Nardini et al. (2015) have revealed that children from 4 to 12 years combine audio-visual information to make spatial decisions, but overall speed and efficiency of MI improves with age. Brandwein et al. (2011) have shown that MI continues to mature in middle childhood, if not beyond this stage of development and found that audiovisual multisensory facilitation of behavior is still clearly immature at 8 years of age and seems to reach mature levels at around 15 years of age. Also Gori et al. (2012) have confirmed these data and found a similar developmental trajectory also for visuo-haptic integration (Gori et al., 2008). About that, Purpura et al. (2017) highlight that visuo-haptic transfer for object recognition matures slowly during school age, although children as young as 4–5 years of age are already able to benefit from this ability.

However, the maturation of visuo-haptic perception is closely involved in adaptation to the environment and in object exploration and recognition and it plays a central role in the early development of body perception, action and memory (Corbetta and Snapp-Childs, 2009). Addabbo et al. (2015) explored the developmental origins of the ability to visually recognize touching gestures involving human body parts and objects. They demonstrated that 3-months-old babies have already a spontaneous preference for the touching hand to-face gesture and that newborns were able to distinguish touching from non-touching gestures when two hands were involved. This result supports the hypothesis of an implicit ability of the human brain to transform the sight of touch into an inner representation of touch and to use visuo-tactile interactions for environmental adaptation in typical and atypical development (Bolognini et al., 2012).

Less is known about audio-haptic integration in children, except that developmental course would seem to be similar to audio-visual and visuo-haptic too (Nava et al., 2016).

These features indicate that multisensory abilities develop over far longer periods in the human brain than in the animal brain and suggest that early experience with cross-modal cues is essential for multisensory development in all species.

## Multisensory stimulation training in patients with visual defect

The interest in the application of MI in neurological and neuropsychological rehabilitation is closely related to the principle that multisensory processes allow for a coherent representation of biological events coming from environment and thus facilitate adaptation to it.

**Figure 1:**
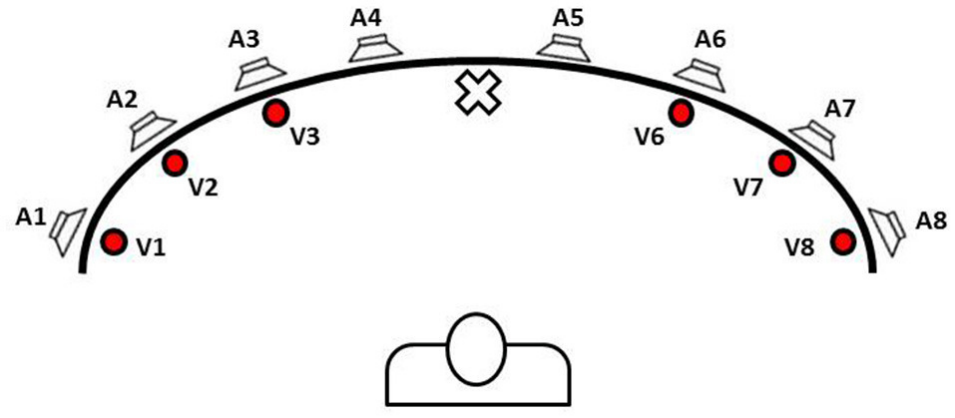
Schematic view of loudspeakers and light displays position in the apparatus for audiovisual stimulation used by Bolognini et al. (2005) and Tinelli et al. (2015). The picture was designed by the authors of this review to explain the multisensory training approach for hemianopic patients. Training was performed with subjects sat in front of the apparatus in a dimly lit and sound-attenuated room and in binocular condition. Subjects were required to look at the fixation point (in the center of the apparatus), and to explore the blind hemifield by shifting their gaze toward visual stimulus, without any head movements. They were instructed to detect the presence of visual target by pressing a button and ignore any auditory stimuli. Fixation was monitored visually by the experimenter standing behind the apparatus, facing the subject. Three different kinds of sensory stimulation were presented: (i) unimodal visual condition; (ii) unimodal auditory condition; and (iii) crossmodal visual–auditory condition. In cross-modal condition, sound could be presented either in the same position as the visual stimulus, i.e., spatially coincident cross-modal condition, or in a different position, i.e., spatially disparate cross-modal condition, at 16 and 32° of nasal or temporal disparity from visual target. Treatment started with 500 ms of stimulus onset asynchrony (SOA) for cross-modal stimuli, i.e., the auditory stimulus preceded the visual target by 500 ms, and SOA was reduced in steps of 100 ms (i.e., 400, 300, 200, and 100 ms) up to the last session of training, in which stimuli were simultaneous (i.e., 0 ms of SOA). Each SOA session terminated when a hit ratio of at least 50% in unimodal visual condition was obtained. Treatment ended when subjects detected more than 50% of the unimodal visual stimuli for three consecutive blocks of trials in the simultaneous presentation of audiovisual stimuli (last SOA session).

Targher et al. (2012) studied audiovisual integration in low-vision adults using a task in which they had to detect the presence of visual stimuli and ignore sound. Results showed a significant effect of synchronous and spatially congruent sounds for visuo-spatial orientation. Similar results were obtained in behavioral studies in hemianopic individuals, in which it has been reported that audio-visual integration can improve some visual functions (Frassinetti et al., 2002; Hairston et al., 2003). In particular, it has been established that the spatially and temporally coincidence of a sound with a visual stimulus can facilitate visual perception and enhance orientation in the blind hemifield of hemianopic patients (Frassinetti et al., 2005). Bolognini et al. (2005) have investigated the possibility of inducing long-lasting improvements in visual field defects by using a training based on systematic audio-visual stimulation of the visual field (for more details on training session see Figure 1). Results indicated progressive ameliorations in visual detections during training and improvement in visual oculomotor strategies, that allowed patients to compensate efficiently for the loss of vision in the damaged hemifield and facilitate visual and spatial orientation. The authors illustrated an interesting transfer of the effect of the treatment to functional measures such as better visual field exploration and improvement in daily life activities which were found to be stable at the 1-month follow-up control session.

Subsequently, Passamonti et al. (2009) verified the specific effects of unimodal stimulation and bimodal stimulation in the enhancement of visual performances, concluding that multisensory training affects several higher order cognitive correlates of visual exploration, such as spatial attention and strategic oculomotor planning. In these two papers (Bolognini et al., 2005; Passamonti et al., 2009), treatment began at least 12 months after onset of lesions, that is when they were stabilized, while Keller and Lefin-Rank (2010) have demonstrated the efficacy of this rehabilitation approach also during the acute phase after the lesions (between 3 and 24 weeks primarily after stroke) in patients with visual field defects. According to the authors, results of audiovisual training exhibited a clear advantage in comparison to a visual exploration training, supporting also the mechanism of “blindsight.” The basic mechanism of this intensive and systematic training could involve the SC in the mediation of residual visual function in hemianopic patients.

Bolognini's protocol was also used by Tinelli et al. (2015). The authors confirmed the efficacy of this rehabilitation approach in children and adolescents with visual field defects due to acquired unilateral brain lesions during childhood. Recently, Jiang et al. (2015) verified the efficacy of a similar training also in hemianopic cats, demonstrating that they become more able of optimizing oculomotor strategies and trasforming visual cues to suitable orientation behaviors. The authors suggest the possibility that multisensory stimulation can promote brain plasticity after the lesion, thus facilitating recovery of visual orientation skills.

Grasso et al. (2016) used electroencephalography measures to assess the brief and long term effects of multisensory stimulation and reported that, after training, patients displayed a significant reduction in posterior-parietal P3 component amplitude in response to stimuli presented in the intact field, reflecting a reduction in attentional resources allocated to the unimpaired field. These results support the idea that audio-visual multisensory stimulation guide long-term plastic changes in hemianopic patients.

In line with encouraging results on the use of audio-visual stimulation in patients with partial visual deficits, in the more recent study of Finocchietti et al. (2017) audio-motor feedback was positively used to improve audio spatial perception in completely blind individuals for optimizing environmental exploration and interaction.

## Environmental enrichment and multisensory stimulation in neurodevelopmental disabilities: perspectives for early intervention

The importance of multisensory stimulation for neurodevelopmental disabilities is supported by several studies showing that an increase in quality and intensity of normal care from the environment exerts profound effects on the CNS and may enhance brain plasticity. For this reason, during experiments with rats, manipulation of standard laboratory conditions was coined Environmental Enrichment (EE), meaning a combination of multisensory/cognitive stimulation, increased physical activity, enhanced social interactions with the aim of eliciting natural explorative behaviors (Baroncelli et al., 2010).

Benefits of EE on synaptic plasticity, visual development and cognitive processes have been investigated in developing, adult, and aging animals and subsequently in animals with several neurologic and genetic disorders (Cancedda et al., 2004; Fischer and Peduzzi, 2007; Xu et al., 2009; Begenisic et al., 2011; Polito et al., 2014).

These findings are very promising for the field of neurorehabilitation. The EE paradigm seems to be a non-invasive and well thought-out model able to enhance the high potential of neuroplasticity through experience and therefore several research groups have tried to investigate if this type of intensive and multisensory stimulation could also have profound positive effects on the human brain.

**Figure 2:**
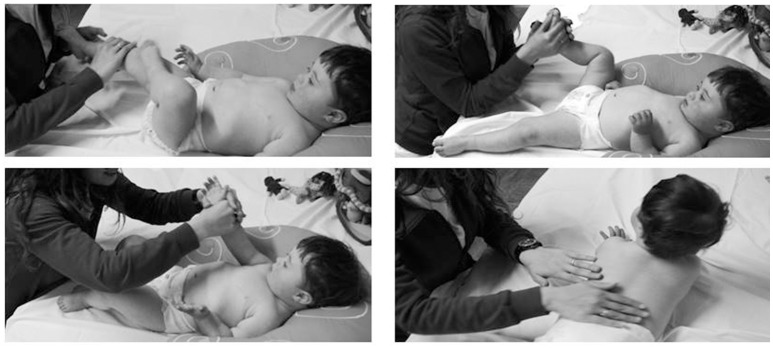
Pictures showing four phases of massage therapy, during which multisensory stimulation on all body parts is provided. Informed consent for the use of the photos was obtained from the parent's subjects featured in the image.

As paradigm of EE in humans, some studies used the “Infant Massage” (IM) that is a standardized practice developed by the International Association of Infant Massage (IAIM®; see Figure 2). IM involves many different ways of touch, movement, interaction and communication and fosters intensive and affective multisensory stimulation. During massage, infants can receive integrated information from the caregiver through different sensory channels (voice, touch, kinesthetic manipulation, facial expression, etc.) and they can focus attention on these complex stimulations for learning and adapting to environment.

IM was tested as early intervention able to influence developmental trajectories in presence of a risk of neurodevelopmental disability. Guzzetta et al. (2009), using IM paradigm, have shown that it affects brain development and visual system maturation in both preterm human infants and in rat pups and suggested that environment acts by modulating the level of endogenous factors such as IGF-1, which regulate brain growth and development of visual cortex. Acceleration of visual acuity development in preterm babies treated with body massage indicates the important role of multisensory stimulation from the environment on brain maturation. Similarly, Purpura et al. (2014) have described positive and profound effects on visual system development (including visual acuity and stereopsis maturation) of multisensory intervention, including IM, in infants with Down Syndrome. Moreover, Guzzetta et al. (2011) have demonstrated that a multisensory stimulation, such as an IM-based therapy, also facilitates the process of maturation of brain electrical activity in low-risk preterm infants, similarly to that observed (*in utero*) in term infants, in particular for the delta and beta band activity. Recently, Gabis et al. (2015) have stated that an early multisensory intervention, including massage performed by parents in neonatal intensive care units, improved language and motor development of preterm children at 2–3 years of age and contributed to significant reductions in parental stress levels. Likewise, massage was included by Medoff-Cooper et al. (2015) within a more complex early intervention in a study that demonstrated the efficacy of the multisensory approach in promoting the organization of sucking in premature infants.

Although the results were interesting and promising, these last studies present some important limitations. Above all, multisensory stimulation was only compared with standard care approaches (i.e., postural, motor, and oral-motor rehabilitation) and it was not compared in a rigorous way with unisensory stimulation (e.g., by randomly assigning participants to interventions). Moreover, it is unclear whether neurobiological mechanisms underlying the IM are related to multisensory stimulation, because this type of intervention was performed during a period in which perceptual abilities are not yet entirely developed and thus could be still not ready to process the rich stimulation provided by a complex and enriched environment. For these reasons, more and well-designed studies are still needed to clarify the short and long term effects of the early multisensory stimulation with IM vs. unisensory stimulation, both in conditions of typical development and in presence of a risk for neurodevelopmental disabilities.

## Conclusion

A growing number of studies have been devoted to highlighting mechanisms that allow for an almost complete recovery of functions in presence of neurodevelopmental disabilities. Taken together, the studies reported in this review suggest that rehabilitation therapies are more likely to be effective if they are based on paradigms of MI and applied as early as possible in order to exploit greater neuronal plasticity.

On the other hand, evidence from MI research demonstrates that experience could produce a functional reorganization, above all in the presence of complex neurodevelopmental disabilities, through noninvasive rehabilitation strategies, specific for both adults and children.

In accordance with these findings, MI may play a great adaptive role and it appears to be essential for developing awareness of our body, space and our body's relationship with the surrounding world by allowing for a decoding of biological and social events. In fact, even in typical development, integration of sensory information plays a key role in the development of speech, non-verbal communication (recognition of gestures, facial expression, etc.) and psychomotor exploration of the environment (Dionne-Dostie et al., 2015). These conclusions suggest that the introduction of a multisensory approach in rehabilitation of children with neurodevelopmental disabilities could be an ecological and evidence-based approach to exploit brain plasticity mechanisms.

To this day, research in the field of evidence-based pediatric rehabilitation is limited by the heterogeneity of interventions used and, thus, by a lack of consistent and reliable data. For this reason, larger and randomized studies are needed to strengthen the generalizability of the findings and to better understand the mechanisms of MI and their use in early interventions for children with neurodevelopmental disability.

## Author contributions

GP, GC, and FT participated in the design, execution, and analysis of the paper entitled “Multisensory-Based Rehabilitation Approach: Translational Insights from Animal Models to Early Intervention of neurodevelopment disabilities” and declare they have seen and approved the final version and that it has neither been published nor submitted elsewhere.

### Conflict of interest statement

The authors declare that the research was conducted in the absence of any commercial or financial relationships that could be construed as a potential conflict of interest.
